# Auxological changes in UK survivors of childhood acute lymphoblastic leukaemia treated without cranial irradiation

**DOI:** 10.1038/bjc.2011.16

**Published:** 2011-02-15

**Authors:** R A L Breene, R M Williams, J Hartle, M Gattens, C L Acerini, M J Murray

**Affiliations:** 1 Department of Paediatric Haematology and Oncology, Addenbrooke's Hospital, Cambridge, CB2 0QQ, UK; 2 Department of Paediatric Endocrinology, University of Cambridge, Cambridge, CB2 0QQ, UK

**Keywords:** acute lymphoblastic leukaemia, auxological, body mass index, childhood, late effects, weight

## Abstract

**Background:**

As most children with acute lymphoblastic leukaemia (ALL) achieve long-term survival, minimising late effects of treatment is a priority. Acute lymphoblastic leukaemia survivors treated historically with protocols including cranial irradiation demonstrate increased weight gain.

**Methods:**

We retrospectively studied all 134 patients treated on the MRC/UKALL97 protocol (without cranial irradiation as standard therapy) at a single centre, with 77 inclusions. Height-, weight- and body mass index (BMI) standard-deviation scores (SDS) were recorded at diagnosis and annually until 3 years out (YO) from end of treatment (EoT); changes across time were explored using a univariate model (significance *P*⩽0.001 to account for multiple comparisons).

**Results:**

Whole-group height SDS was lower from 1 year into treatment until 2 YO, whereas weight- and BMI-SDS remained higher until 3 YO. In females, height-SDS was lower until EoT, but higher weight- and BMI-SDS persisted until 3 YO. In males, height-SDS was lower at EoT and at 2 YO; differences in BMI-SDS had resolved by 2 YO. By WHO criteria, more patients were overweight or obese at 3 YO than at diagnosis (*P*=0.01).

**Conclusion:**

Survivors of childhood ALL, particularly females, exhibit adverse changes in height-, weight- and BMI-SDS, which arise during treatment and persist into follow-up. Patients should be supported with appropriate dietary and lifestyle advice during ALL treatment and follow-up, which may minimise these changes and reduce associated long-term morbidity.

Acute lymphoblastic leukaemia (ALL) is the most common childhood cancer. As over 80% of patients now achieve 10-year overall survival (Mitchell *et al*, 2010), attention now focuses on minimising sequelae of the condition and its treatment. Increased obesity has been reported amongst survivors of childhood ALL, with cranial irradiation as a major risk factor (Odame *et al*, 1994), with one study demonstrating obesity in 50% of ALL survivors at final height (Didi *et al*, 1995). The most recent UK report, which included a cohort of unirradiated patients treated on the MRC/UKALL XI protocol, demonstrated raised body mass index standard deviation scores (BMI-SDS) in both males and females, from diagnosis to the end of treatment (EoT), but this increase did not persist to last follow-up (Craig *et al*, 1999).

More recent treatment protocols have replaced cranial irradiation with intrathecal chemotherapy as standard central nervous system disease prophylaxis and treatment. Published reports on the auxological effects of these changes are conflicting. An increased incidence of overweight and obesity is reported amongst some US cohorts (Baillargeon *et al*, 2007; Chow *et al*, 2007). However, a retrospective report from the US Childhood Cancer Survivor Study did not identify any BMI increase in ALL survivors treated with chemotherapy alone, although self-reported heights and weights were used and adult thresholds of overweight and obese used (Garmey *et al*, 2008).

There have been no reports on auxological parameters in a UK cohort treated on a contemporary treatment protocol without cranial irradiation as standard therapy. We therefore performed a retrospective longitudinal single-centre study to evaluate the relative contributions of height and weight to BMI at different time points during treatment and follow-up in patients treated on the MRC/UKALL97 protocol.

## Patients and methods

Retrospective case-note analysis was performed on all children with ALL enrolled on the MRC/UKALL97 protocol at Addenbrooke's Hospital, Cambridge, UK between 1997 and 2003 and aged 1–16 years at diagnosis. Anonymised demographic and treatment data was recorded between January and April 2008. Height and weight measurements at ALL diagnosis and annual time points until 3 years out (YO) from EoT were collected. Body mass index was calculated at each time point using the standard formula: weight (kg)/height (m)^2^. Height-, weight- and BMI-SDS were calculated relative to British age- and population-referenced normative data (Cole, 1990). Exclusion criteria were relapse, death, irradiation (cranial or testicular), bone marrow transplant or other co-existing condition affecting long-term growth (namely chronic liver disease, which was presumed to occur as a result of use of 6-thioguanine during ALL treatment).

Analyses were performed using SPSS version 10 (SPSS (UK) Limited, IBM, Woking, UK). Changes in height-, weight- and BMI-SDS across time from diagnosis were explored using a univariate model including each of these as the dependent variable, incorporating both the time from diagnosis and subject identification. *Post-hoc* analysis for time was performed using Dunnett's two-sided test. To account for multiple comparisons, a *P*-value ⩽0.001 (two-tailed) was considered significant. Between-group differences in height-, weight- and BMI-SDS changes between diagnosis and 3 YO by gender, diagnostic white cell count (WCC; < or ⩾50 × 10^9^ l^−1^), MRC/UKALL97 treatment regimen (A/B/high risk) and steroid randomisation (prednisolone/dexamethasone) were performed using one-way analysis of variance for patients with complete data sets to 3 YO. Overall frequency of overweight (BMI-SDS 1.3–2.3) and obesity (BMI-SDS >2.3) in patients at diagnosis and 3 YO were determined according to standard WHO criteria (Cole *et al*, 2000) and between-group differences examined using *χ*^2^-test (*P*<0.05 significant).

## Results

### Study patients/demographics

From a total of 134 patients enrolled, 57 (42%) were excluded: 28 died, 9 relapsed, 7 received irradiation (6 cranial, 1 testicular), 1 patient underwent bone marrow transplant, 9 developed chronic liver disease, 2 had insufficient auxological data at diagnosis and 1 patient transferred to adult care. Of the 77 patients included, 37 (48%) were male and 40 (52%) were female. Median (range) age (year) at diagnosis was 4.6 (1.1–15.9), with no gender difference (females 4.3 (1.1–13.1), males 5.6 (1.5–15.9)). Median age at 3 YO was 9.8 years in females and 11.8 years in males, giving an overall median of 10.1 (6.5–20.1). At the time of data capture, 13 of the 77 patients had not reached 3 YO and a further 11 either did not have measurements recorded or were lost to follow-up. Distribution of ethnic groups reflected that of the local population (87% White British).

### Disease/treatment characteristics

Of the 77 patients included, 67 patients (87%) had low WCC at diagnosis (<50 × 10^9^ l^−1^) and 10 (13%) were high count (⩾50 × 10^9^ l^−1^). In total, 52 patients (67.5%) were treated on MRC/UKALL97 protocol Regimen-A, 19 patients (24.7%) on Regimen-B and 6 patients (7.8%) on the high-risk protocol, consistent with overall UK figures. A total of 30 patients (39%) received prednisolone and 47 received (61%) dexamethasone – the unequal distribution attributable to dexamethasone becoming standard therapy during the MRC/UKALL97 study because of its superior event-free survival (Mitchell *et al*, 2005). Most male patients received 2 years of maintenance ALL therapy (i.e., approximately 3 years treatment in total), and therefore received a greater cumulative dose of steroid treatment than females, who only received 1 year of maintenance.

### Whole group and gender subgroup comparisons

**Figure 1:**
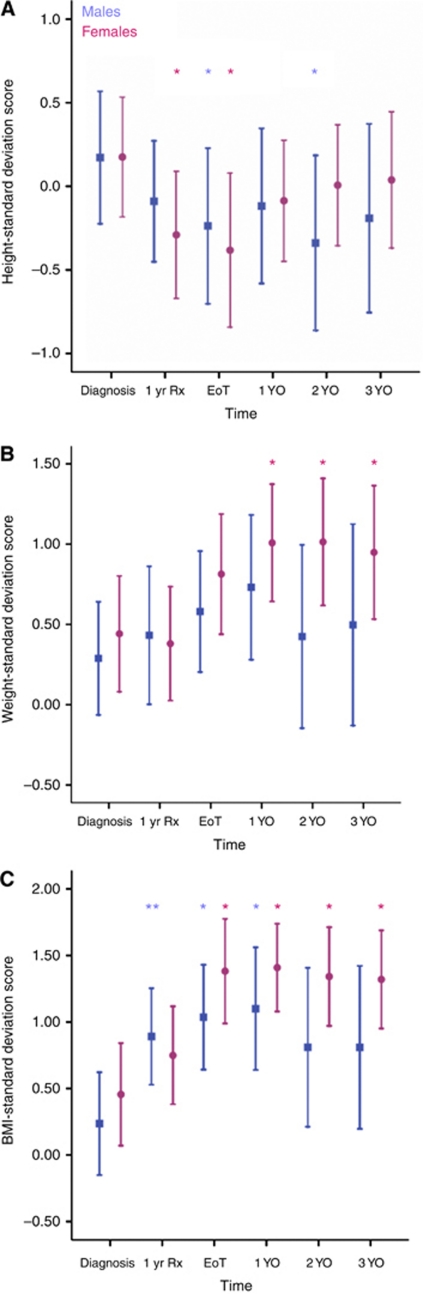
Mean height- (**A**), weight- (**B**) and BMI- (**C**) SDS from diagnosis to 3 years out (YO) from end of treatment (EoT) by gender subgroup (95% confidence intervals; CIs). Results for males are depicted by squares; for females by circles. 1 yr Rx=1 year of treatment. Asterisks denote significant differences in height-, weight and BMI-SDS at that time point when compared with values at diagnosis. ^*^P<0.0001; ^**^P=0.001.

Adjusted mean height-, weight- and BMI-SDS for whole group and gender subgroups at diagnosis, 1 year into treatment, EoT and yearly intervals to 3 YO are shown in Table 1. Height: whole-group mean height-SDS was reduced from 1 year into treatment to 2 YO. For the male subgroup, mean height-SDS was reduced at EoT and 2 YO, whereas for females the scores were reduced at 1 year into treatment and EoT (Figure 1A). Weight: whole-group mean weight-SDS was higher from EoT to 3 YO. When analysed by gender, mean weight-SDS for males was unchanged. Females showed raised mean weight-SDS at all time points from 1 YO until 3 YO (Figure 1B). Body mass index: whole-group mean BMI-SDS was higher from 1 year into treatment and still raised at 3 YO. In males, the mean BMI-SDS was elevated from 1 year into treatment until 1 YO. In the female subgroup, mean BMI-SDS was elevated from EoT and at all time points to 3 YO (Figure 1C).

### Between-group comparisons for BMI

There were no differences in changes in BMI-SDS from diagnosis to 3 YO in direct comparisons between groups for WCC at diagnosis, treatment regimens, steroid agent used or gender. This may reflect the relatively small numbers in each group.

### Overall frequency of overweight/obesity

More patients were overweight or obese at 3 YO than at diagnosis (25 out of 53 (47.2%) *vs* 23 out of 77 (29.9%), respectively; *P*=0.01) (Table 2).

## Discussion

Despite the lack of cranial irradiation in current treatment protocols, we have shown that UK survivors of childhood ALL have increases in BMI-SDS, which persist well into follow-up. Our data extend previous historical observations. Craig *et al* (1999) demonstrated that both male and female unirradiated ALL patients had increased BMI-SDS at EoT in a UK cohort, although such changes were no longer significant at last follow-up. An increased BMI-SDS at EoT was also demonstrated in a US cohort treated without cranial irradiation, but no gender difference was identified, possibly because patients were not followed up beyond EoT (Baillargeon *et al*, 2007). Our findings reveal that females are at particular risk for increases in BMI-SDS and that such changes persist from EoT until at least 3 YO. These data are consistent with a US study, which not only identified increased BMI at EoT but also that the proportion of patients overweight or obese was unchanged 2–3 years later, with females at increased risk of such findings (Chow *et al*, 2007).

Our systematic examination of height- and weight-SDS in this study enables conclusions to be drawn about the relative contribution of each to BMI in the whole group and in gender subgroup analyses. Early BMI increases reflect reduced height-SDS, whereas weight-SDS remains static. These changes are likely due to acute suppressive effects of steroid therapy and leukaemic illness *per se* on height velocity, with absent weight gain common initially because of treatment intensity. Subsequent elevation of BMI-SDS appears secondary to weight gain, with females particularly susceptible. In males, BMI-SDS changes are transient, recovering by 2 YO. As males do not demonstrate weight-SDS changes, increased BMI reflects slow height-SDS recovery. In females, BMI increases do not appear until EoT, but then persist until 3 YO, and appear predominantly secondary to effects on weight, as height recovers by 1 YO. The weight gain observed in females is not attributable to total steroid dose received, as males, who receive greater cumulative doses as a result of 2 years maintenance therapy, did not display such findings. However, the slower height recovery seen in males is consistent with the effects of longer steroid treatment on linear growth. Our findings suggest a sexually dimorphic phenomenon, with the relative contributions to persistently increased BMI from increased dietary intake, altered metabolism and/or reduced physical activity in females yet to be determined. Of note, females are more likely to have reduced physical activity during cancer treatment and follow-up (Winter *et al*, 2010), with female ALL survivors having also been identified as at risk of poor physical fitness (Jarvela *et al*, 2010).

Survivors of childhood cancer are already at risk of adverse long-term health outcomes, such as increased cardiovascular risk with higher rates of myocardial infarction (Mulrooney *et al*, 2009), and females appear to be at greater risk than their male counterparts (Armstrong *et al*, 2007). Although small BMI-SDS reductions have been demonstrated to lead to improvements in cardio-metabolic risk in obese adolescents (Ford *et al*, 2010), longitudinal cohort studies such as the Bogalusa Heart Study have demonstrated that childhood overweight persists into adulthood and is associated with increased frequency of features of the metabolic syndrome in adulthood (Camhi *et al*, 2010). These findings suggest that intervention strategies, targeting both patients’ dietary and lifestyle choices, might be required to prevent weight gain and physical inactivity developing during ALL treatment and follow-up.

We recognise that our study has a number of limitations. First, as a retrospective study, we did not have a control group for comparison. The inclusion of longitudinal data from healthy children, measured against the same reference data would strengthen our observations. However, the children included in our report were measured serially with SDS for auxological parameters calculated against current UK standard reference data (Cole *et al*, 2000). The frequency of overweight and obese in our cohort as determined by WHO standards approaches 50% at 3 YO. The most recent reports from the UK national child measurement programme (using the same reference data) report prevalence rates of overweight and obesity (school year 6, 2009/2010) in our region of 26–32% (SEPHO, 2010), similar to the prevalence in our cohort at diagnosis (29.9%). It is therefore likely that the increased frequency we describe is real. Second, given that the overall median age of patients was still only 10.1 years at 3 YO, it will be important to continue to follow this cohort through puberty to final height. As dietary intake and physical activity were not measured in this study, it will also be important in the future to prospectively study the impact of targeted interventions on these parameters, and other more sophisticated auxological indices of overweight.

In conclusion, childhood survivors of ALL treated without cranial irradiation are at risk of BMI increases, which persist into follow-up, with females appearing particularly susceptible. Through targeted intervention strategies we may aim to minimise the associated long-term morbidity and mortality in this patient group.

## Figures and Tables

**Table 1:** Height-, weight- and BMI-SDS in whole group and male and female subgroups from diagnosis to 3 years out (YO) from end of treatment (EoT)

		**Whole group**			**Male subgroup**			**Female subgroup**		
**Time point**		**Mean (95% CI)**	***N***	***P*-value**	**Mean (95% CI)**	***N***	***P*-value**	**Mean (95% CI)**	***N***	***P*-value**
Height-SDS	Diagnosis	0.17 (0.09–0.26)	77		0.17 (0.04–0.30)	37		0.18 (0.07–0.28)	40	
	1 year into treatment	−0.27 (−0.36 to −0.18)	69	**<0.0001**	−0.21 (−0.35 to −0.06)	32	0.03	−0.32 (−0.43 to −0.21)	37	**<0.0001**
	End of treatment	−0.31 (−0.40 to −0.22)	68	**<0.0001**	−0.27 (−0.41 to −0.13)	32	**<0.0001**	−0.35 (−0.47 to −0.24)	36	**<0.0001**
	1 year post EoT (1 YO)	−0.09 (−0.18 to 0.00)	66	**<0.0001**	−0.06 (−0.20 to 0.08)	31	0.02	−0.09 (−0.20 to 0.03)	35	0.005
	2 years post EoT (2 YO)	−0.04 (−0.14 to 0.06)	58	**<0.0001**	−0.09 (−0.26 to 0.09)	23	**<0.0001**	−0.01 (−0.13 to 0.10)	35	0.13
	3 years post EoT (3 YO)	0.01 (−0.09 to 0.11)	53	0.004	−0.06 (−0.25 to 0.13)	20	0.006	0.05 (−0.07 to 0.17)	33	0.31
										
Weight-SDS	Diagnosis	0.37 (0.26–0.48)	77		0.29 (0.12–0.46)	37		0.44 (0.30–0.59)	40	
	1 year into treatment	0.41 (0.30–0.52)	69	0.99	0.45 (0.27–0.62)	32	0.671	0.38 (0.24–0.53)	37	0.97
	End of treatment	0.74 (0.62–0.85)	68	**<0.0001**	0.62 (0.44–0.80)	32	0.078	0.85 (0.70–1.00)	36	0.003
	1 year post EoT (1 YO)	0.83 (0.72–0.96)	66	**<0.0001**	0.72 (0.52–0.91)	31	0.003	0.96 (0.80–1.11)	35	**<0.0001**
	2 years post EoT (2 YO)	0.83 (0.70–0.96)	58	**<0.0001**	0.61 (0.39–0.83)	23	0.789	1.00 (0.84–1.15)	35	**<0.0001**
	3 years post EoT (3 YO)	0.78 (0.65–0.92)	53	**<0.0001**	0.55 (0.31–0.79)	20	0.471	0.95 (0.79–1.11)	33	**<0.0001**
										
BMI-SDS	Diagnosis	0.35 (0.20–0.50)	77		0.24 (0.01–0.46)	37		0.46 (0.27–0.64)	40	
	1 year into treatment	0.81 (0.65–0.97)	69	**<0.0001**	0.87 (0.62–1.13)	32	**0.001**	0.77 (0.57–0.96)	37	0.13
	End of treatment	1.29 (1.13–1.45)	68	**<0.0001**	1.11 (0.85–1.36)	32	**<0.0001**	1.46 (1.26–1.66)	36	**<0.0001**
	1 year post EoT (1 YO)	1.21 (1.05–1.37)	66	**<0.0001**	1.05 (0.79–1.31)	31	**<0.0001**	1.36 (1.15–1.56)	35	**<0.0001**
	2 years post EoT (2 YO)	1.14 (0.96–1.31)	58	**<0.0001**	0.86 (0.56–1.17)	23	0.01	1.34 (1.14–1.55)	35	**<0.0001**
	3 years post EoT (3 YO)	1.04 (0.85–1.22)	53	**<0.0001**	0.77 (0.43–1.10)	20	0.02	1.24 (1.03–1.45)	33	**<0.0001**

Abbreviations: BMI=body mass index; *N*=number of patients included in the analysis at the stated time-point; SDS=standard deviation score.

Adjusted means (95% confidence intervals; CI) are listed. Significant *P*-values are displayed in bold.

**Table 2:** Frequency of overweight and obese at diagnosis and 3 years from end of treatment (3 YO)

**WHO group**	**At diagnosis (*N*=77)**	**At 3 YO (*N*=53)**	***P*-value**
Healthy (BMI-SDS <1.3)	54 (70.1%)	28 (52.8%)	Not significant
Overweight (BMI-SDS 1.3–2.3)	21 (27.3%)	16 (30.2%)	Not significant
Obese (BMI-SDS >2.3)	2 (2.6%)	9 (17.0%)	Not significant
Overweight or obese (BMI-SDS >1.3)	23 (29.9%)	25 (47.2%)	0.01

Abbreviations: BMI=body mass index; *N*=number of patients included in the analysis is not the stated time-point; SDS=standard deviation score.

Difference assessed by *χ*^2^-test; *P*<0.05 significant.
